# Chemical Characterization and Evaluation of Antimicrobial Properties of the Wild Medicinal Mushroom *Ganoderma lucidum* Growing in Northern Moroccan Forests

**DOI:** 10.3390/life13051217

**Published:** 2023-05-19

**Authors:** El Hadi Erbiai, Benoutman Amina, Abbassi Kaoutar, Rabah Saidi, Zouhaire Lamrani, Eugénia Pinto, Joaquim C. G. Esteves da Silva, Abdelfettah Maouni, Luís Pinto da Silva

**Affiliations:** 1 Biology, Environment, and Sustainable Development Laboratory, ENS, Abdelmalek Essaadi University, Tetouan 93000, Morocco; elhadi.erbiai@etu.uae.ac.ma (E.H.E.); amina.benoutman@etu.uae.ac.ma (B.A.); kaoutar.abbassi@etu.uae.ac.ma (A.K.); r.saidi@uae.ac.ma (R.S.); zh.amrani@yahoo.fr (Z.L.); amaouni@uae.ac.ma (A.M.); 2 Chemistry Research Unit (CIQUP), Institute of Molecular Sciences (IMS), Department of Sciences, Environment and Territorial Planning, Faculty of Sciences, University of Porto, Rua do Campo Alegre s/n, 4169-007 Porto, Portugal; jcsilva@fc.up.pt; 3 Laboratory of Microbiology, Biological Sciences Department, Faculty of Pharmacy, University of Porto (FFUP), 4050-313 Porto, Portugal; epinto@ff.up.pt; 4 Interdisciplinary Centre of Marine and Environmental Research (CIIMAR), University of Porto, 4450-208 Matosinhos, Portugal

**Keywords:** *Ganoderma lucidum*, medicinal mushroom, Moroccan mushroom, bioactive compounds, phenolic compounds, antioxidant activity, dermatophytes, antimicrobial activity

## Abstract

*Ganoderma lucidum* is an extensively famous medicinal mushroom distributed worldwide. Despite being widely grown in Moroccan forests, there are no studies on its nutritional, nutraceutical and pharmaceutical values. Herein, the objective of this study was to investigate the chemical characterization and antimicrobial properties of *G. lucidum* methanolic extract. Total phenolic, flavonoid, tannin, ascorbic acid and carotenoid contents were determined by spectrophotometry. The results revealed that the most prevalent bioactive compounds were phenolics and flavonoids, with total values of 154.60 mg GAE/g of dry methanolic extract (dme) and 60.55 mg CE/mg of dme, respectively. A GC–MS analysis identified 80 biologically active molecules, which were mainly divided into the following major groups: sugars (49.49%), organic acids (8.89%), fatty acids (7.75%), amino acids (7.44%), steroids (7.32%), polyphenols (5.92%), and others (13.16%). Additionally, 22 individual phenolic compounds were identified and quantified using HPLC–MS, with emphasis on kaempferol (1714 µg/g of dry weight (dw)), apigenin (1955 µg/g dw) and quercetin (947.2 µg/g dw). The methanolic extract of *G. lucidum* indicated strong antioxidant capacity by means of the following: DPPH radical-scavenging activity (53.7 µg/mL), β-carotene/linoleate assay (43.75 µg/mL), and reducing power assay (76.62 µg/mL). Furthermore, the extract exhibited potent antimicrobial properties against seven human pathogenic microorganisms, including two bacteria and five fungal strains, at concentrations ranging from 1 to 16 mg/mL. The most sensitive pathogen was *Epidermophyton floccosum* (MIC = MFC = 1 mg/mL), while *Aspergillus fumigatus* was the most resistant one (MIC = 16 mg/mL and MFC ≥ 16 mg/mL). Overall, our findings demonstrated valuable nutritional and bioactive compound attributes, and potent antioxidant and antimicrobial properties, of *G. lucidum* growing in Moroccan forests. Moreover, these findings suggest that the Moroccan mushroom can be extremely useful for the food and medicinal industries to positively affect socioeconomic status.

## 1. Introduction

Many infectious diseases worldwide, especially in developing countries, are generally caused by pathogenic microorganisms, such as bacteria, fungi, viruses, and parasites, leading to mortality and morbidity [1,2]. Among these pathogens are dermatophytes, a group of filamentous fungi that infect the skin, nails, and hair stratum corneum, causing dermatophytosis [3]. Mushrooms, as a natural resource for different bioactive and nutraceutical compounds, have already demonstrated strong activities against numerous infectious diseases [4,5,6]. Thus, they can be significant in the discovery of new compounds able to combat multiple pathogenic microorganisms.

*Ganoderma lucidum* (Curtis) P. Karst. (1881), is a basidiomycete mushroom, commonly known as “Lingzhi” in China and as “Reishi” in Japan, which belongs to the *Ganodermataceae* family and *Polyporales* order, and grows in temperate and subtropical locations in America, Asia, Europe and Africa [2,7]. *G. lucidum* fruiting bodies first grow parasitically on living hosts and, then, saprophytically on dead hosts, typically found at the base of, or, rarely, on the roots of, a wide range of deciduous trees, including *Acer*, *Celtis*, *Quercus*, *Salix*, *Fraxinus*, or *Ulmus* [7]. Being considered an edible and medicinal resource, *G. lucidum* has been widely used for centuries as a herb and as an important ingredient in traditional medicine in East Asian countries for the prevention and treatment of many human diseases, including microbial diseases, cancer, diabetes, hepatopathy, hepatitis, arthritis, hypertension, neurasthenia, allergies [5,8,9,10,11,12]. Nowadays, *G. lucidum* is commercialized around the world for use as a powder, tea, and dietary supplement [11,13]. Over 400 bioactive molecules have been detected and identified from fruiting bodies, spores and mycelia of *G. lucidum*, including polysaccharides, steroids, triterpenoids, sterols, fatty acids, alkaloids, amino acids, proteins, nucleosides, and others. These biologically active compounds have several medicinal properties, including the following: anti-inflammatory, antioxidant, antifungal, antibacterial, antiviral, antitumor, antiaging, anti-atherosclerotic, anti-diabetic, immunomodulatory, hypolipidemic and others [2,11,13,14]. Generally, polysaccharides and triterpenoids from *G. lucidum* are the main compounds with medicinal properties [13,15,16]. Nonetheless, as far as we know, there are few studies on the non-volatile compounds of *G. lucidum* fruiting body alcoholic extracts and their antimicrobial activity against human pathogens, particularly dermatophytes. 

In Morocco, *G. lucidum* is widely found on, and/or under, *Quercus* species (including, *Q. ilex*, *Q. suber*, and *Q. faginea*), *Abies pinsapo*, and *Cedrus atlantica* and on holly tree roots. It can be found in the following locations: Harcha, Oulmes, Lalla Mimouna forests and exotic gardens of Bouknadel (Rabat-Sale-Kenitra region), Cherf El Akab and Diplomatic forests (Tangier), Larache, Djebel Timellatine (Targuist, Rif), Bab-Er-Rouida forest (Bab Taza, Rif), Marcha forest (Monts Zaïans, Rif), Azrou forest and Bab-bou-Idir forest (Taza) [17,18,19,20,21,22]. However, to the best of our knowledge, there are no studies on the chemical characterizations and biological properties of *G. lucidum* growing in Morocco. 

Therefore, in the present study, the *G. lucidum* fruiting body was collected from northwestern Morocco and chemically analyzed in order to determine its bioactive compounds, to characterize its polyphenols, by means of high-performance liquid chromatography–mass spectrometry (HPLC–MS), and to identify its biologically active molecules, by means of gas chromatography–mass spectrometry (GC–MS). Moreover, the methanolic extract of the studied mushroom was evaluated for its antioxidant activity (DPPH, β-carotene/linoleic acid and ferric ion reducing power test) and its antimicrobial properties against seven human pathogenic microorganisms, including two bacteria, one yeast, one *Aspergillus* and three dermatophytes. Our findings demonstrated valuable nutritional and bioactive compound attributes and potent antioxidant and antimicrobial properties of *G. lucidum* growing in Moroccan forests.

## 2. Materials and Methods

### 2.1. Chemical Reagents and Standards

Folin–Ciocalteu phenol reagent, linoleic acid, sodium carbonate, sodium nitrite, meta-phosphoric acid, sodium hydroxide, 2,6-Dichloroindophenol sodium salt hydrate, alkane standards (C_8_–C_20_ and C_21_–C_40_), dimethyl sulfoxide (DMSO), gentamicin, l-ascorbic acid, MOPS, Tween 40, N,O-Bis(trimethylsilyl)trifluoroacetamide (BSTFA), β-carotene, vanillin reagent, individual phenolic standards (apigenin, apigenin 7-glucoside, caffeic acid, catechin, chlorogenic acid, cinnamic acid, ellagic acid, ferulic acid, gallic acid, isorhamnetin, kaempferol, luteolin, luteolin 7-glucoside, methylparaben, naringin, *p*-coumaric acid, *p*-hydroxybenzoic acid, protocatechuic acid, quercetin, rosmarinic, rutin, salicylic, syringic, vanillic acid, and vanillin), iron (III) chloride and (±)-6-Hydroxy-2,5,7,8-tetramethylchromane-2-carboxylic acid (Trolox) were obtained from Sigma-Aldrich, Co., (St. Louis, MO, USA). Aluminum chloride, acetonitrile, hydrochloric acid fuming 37%, sodium chloride, pyridine and ethyl acetate were purchased from Merck KGaA (Darmstadt, Germany), and PDA (Potato Dextrose Agar) was obtained from Difco Laboratories (Detroit, MI, USA). Acetone, hexane, and n-hexane were acquired from CABLO ERBA Reagent, S.A.S (Val de Reuil Cedex, France), and RPMI-1640 was from Biochrom AG (Berlin, Germany). MHB (Mueller–Hinton broth), SDA (Sabouraud dextrose agar), and MHA (Mueller–Hinton agar) were purchased from BioMérieux (Marcy L’Étoile, France), and DPPH (2,2-diphenyl-l-picrylhydrazyl) was obtained from Alfa Aesar (Ward Hill, MA, USA). Voriconazole was bought from Pfizer and terbinafine from NOVARTIS. Methanol and all other solvents were purchased from Honeywell (St. Muskegon, MI, USA).

### 2.2. Macrofungi Material

*G. lucidum* fruiting body was collected near the root of the living tree, *Pinus halepensis*, at the Biological and Ecological Interest Site (SIBE) of Koudiat Taifour in northwestern Morocco (35°40′45.4″ N 5°17′36.3″ W, 180 m altitude, thermo-Mediterranean vegetation level, siliceous substrate of shale, and subhumid bioclimatic level in temperate winter) in February 2018. Mushroom identification used morphological and ecological characterizations, and followed two determination keys [23,24]. The voucher specimen was deposited at the BEDD laboratory herbarium, ENS, Abdelmalek Essaadi University, Tetouan, Morocco. *G. lucidum* was cultivated on a PDA medium at 25 °C, and the pure mycelium obtained was perfectly preserved for future use. The basidiocarps were cleaned, cut, weighed, air-dried, and ground to a fine powder (20 mesh).

### 2.3. Methanolic Extract Preparation

The preparation of crude methanolic extracts of the *G. lucidum* fruiting body was assayed following an extraction method described previously by Barros et al. [25], with slight modifications. Briefly, 0.5 g of the sample powder (20 mesh) was extracted by shaking with 20 mL of methanol and, subsequently, filtered through Whatman N °4 paper. The filtered residue was reextracted twice, following the same process. The combined solutions were evaporated under reduced pressure at 40 °C to dryness. Afterwards, the methanolic extracts were weighed and stored at −81 °C.

### 2.4. Bioactive Compounds Determination

Bioactive compound contents existing in the *G. lucidum* basidiocarps were determined spectrophotometrically using the same conditions, equipment and methods of our published work [26].

Total phenolic compound content was estimated by the Folin–Ciocalteu method. Concisely, an extract of methanolic solution (1 mL), sodium carbonate solution (4 mL) and Folin–Ciocalteu reagent (5 mL) were mixed and allowed to stand for 30 min at 40 °C in the dark. The absorbance was recorded at 765 nm using an UV-Visible spectrophotometer. The total phenolic content was expressed as milligrams of gallic acid equivalents (GAEs) per gram of dry methanolic extract (dme).

Total flavonoid content determination was carried out according to an aluminum chloride colorimetric assay, which can detect flavonoids in a group of flavonols and flavones. The absorbance measurement was taken at 510 nm. The total flavonoid content was expressed as milligrams of (+)-catechin equivalents (CEs) per gram of dme.

The estimation of total ascorbic acid content in the *G. lucidum* fruiting body was done by mixing meta-phosphoric acid (1%) extract and the reagent 2,6 dichlorophenolindophenol. This method detected ascorbic acid when absorbance was recorded at 515 nm. The ascorbic acid content was expressed as mg of l-ascorbic acid equivalents (AAEs) per gram of dw.

Total tannin content was performed using the Vanillin–HCL method. The color-developed absorbance was measured at 500 nm. The total tannin content was expressed as mg of CEs/g of dme.

Total contents of β-carotene and lycopene were determined using a method that mixed acetone–hexane (4:6) and methanolic extract. The solution absorbance (A) was measured at 453, 505, 645 and 663 nm. The carotenoids contents were calculated using the following equations: *β-Carotene* (mg/100 mL) = [(0.216 A_663_) − (0.304 A_505_) + (0.452 A_453_)]; *Lycopene* (mg/100 mL) = [(0.0458 A_663_) + (0.372 A_505_) − (0.0806 A_453_)].

### 2.5. Biomolecules Analysis by GC–MS

The gas chromatography–mass spectrometry (GC–MS) analysis of biologically active molecules present in the methanolic extract of the medicinal mushroom *G. lucidum* fruiting body from northern Morocco was performed using the same conditions, equipment and process described by Erbiai et al. [27]. Briefly, the derivatized methanolic extract solution was analyzed by the GC–MS system and automatic injector. The GC separation was operated with a TG5-MS capillary column (60 m × 0.25 mm i.d.; 0.25 µm film thickness) with a non-polar stationary phase. Identification of biomolecules was determined using Kovats retention indices (RIs) relative to alkanes (C_8_–C_20_ and C_21_–C_40_) of authentic molecules and with the spectral data acquired from the databases of the National Institute Standard and Technology (NIST) and PubChem Libraries of the corresponding compounds. Data was acquired using Software Thermo Xcalibur^TM^ 2.2 SP1.48, and data was analyzed using NIST MS Search 2.2 Library 2014.

### 2.6. Individual Polyphenol Analysis by HPLC–MS

Individual phenolic compounds existing in *G. lucidum* were extracted and characterized by using the same HPLC equipment, conditions and procedure described in our published study [26]. In brief, the polyphenolic extract was analyzed by means of high-performance liquid chromatography–mass spectrometry (HPLC–MS). Chromatographic separation was performed using Acclaim™ 120 reverse phase C18 columns (3 µm 150 × 4.6 mm), thermostatted at 35 °C, and peaks were detected at 280 nm as the preferred wavelength. The mobile phase contained 1% acetic acid and 100% acetonitrile. Detection was carried out in a photodiode array detector (PDA), with 280 nm being the preferred wavelength.

The LC–MS analysis was performed using an LC quaternary Plus pump, coupled to a Finningan LCQ Deca XP MAX mass detector with an electrospray ionization (ESI) source and an ion trap quadrupole (Thermo Scientific, Waltham, MA, USA). Regarding MS detection, nitrogen served as the sheath gas (40 psi) and auxiliary gas (15 psi), and ultra-high pure helium as the collision gas (medium). The analysis was performed in negative ion mode with a mass range of *m*/*z* 120–1000. The system was operated as follows: capillary temperature of 300 °C; source voltage of 5 kV; capillary voltage of −30 V; tube lens offset of −60 V.

The individual polyphenols were identified according to their UV-Vis spectra and by their mass spectra and retention times in comparison with authentic commercial standards. Quantification was based on the peak areas registered at 280 nm and calibration curves acquired from the standard of each compound. The phenolic compound contents were expressed in µg per gram of dry weight (dw). The LC–MS functions and all the data collected and processed were carried out using Xcalibur^TM^ 2.2 SP1.48 software (Thermo Scientific, Waltham, MA, USA).

### 2.7. Antioxidant Properties Evaluation

The evaluation of the antioxidant properties of the *G. lucidum* fruiting body methanolic extracts was accomplished spectrophotometrically using the following three different methods previously described by Heleno et al. [28]: DPPH, β-carotene/linoleic acid and ferric ion-reducing power test. Graphs derived from the antioxidant studies were used to calculate the extract/standard concentration causing 50% of the antioxidant property or 0.5 of absorbance (EC_50_). The reference standard used in this study was Trolox. The strongest antioxidant activity of the sample corresponded to the lowest EC_50_ value.

The DPPH radical-scavenging activity (RSA) of the mushroom extracts was carried out using the stable radical 1.1-diphenyl-2-picrylhydrazyl (DPPH^•^). Thus, mixtures of methanolic extract (0.3 mL) of different concentrations and DPPH solution (2.7 mL) were allowed to stand for 30 min at room temperature, and, subsequently, measurement of absorbance was taken at 517 nm using an UV-Vis spectrophotometer. The antiradical activity was calculated as a percentage of DPPH discoloration in the following equation: RSA (%) = [(A_DPPH_ − A_Sample_)/A_DPPH_] × 100, where A*_DPPH_* is the absorbance of the DPPH solution, while A*_Sample_* is the absorbance of the test extract.

Regarding the anti-lipid peroxidation activity, the antioxidant property of *G. lucidum* extract was evaluated by using the β-carotene/linoleic acid system, which, based on the capacity of compounds exiting in the extract to neutralize the linoleic acid-free radicals, avoids β-carotene bleaching. The absorbance of oxidation of β-carotene emulsion was taken at zero-time and 120 min at 470 nm against a blank. The β-carotene/linoleic acid bleaching inhibition was determined using the formula: (%) = (β-carotene content at 120 min of the assay/ β-carotene content at zero-time) × 100.

For the ferric ion reducing power test, the ferricyanide/Prussian blue method was used to evaluate the capacity of antioxidant compounds in the *G. lucidum* extract to convert Fe^3+^ into Fe^2+^. The solution absorbance was determined spectrophotometrically at 690 nm against the blank.

### 2.8. Evaluation of Antimicrobial Properties

The Clinical and Laboratory Standard Institute-CLSI, (M07-A8, bacteria; M27-A3, yeasts; M38-A2, filamentous fungi) broth microdilution method was chosen to assess the antimicrobial activities of *G. lucidum* methanolic extract. This technique determines the minimum inhibitory concentration (MIC) and, then, the minimum bactericidal/fungicidal concentration (MBC/MFC). Gentamicin, terbinafine and voriconazole were included as antimicrobial agents used for therapeutics [29,30,31,32].

#### 2.8.1. Microorganisms and Culture Media

Two bacterial and five fungal human pathogenic microorganisms were employed to test the antimicrobial properties of the *G. lucidum* extracts. The antibacterial property was tested against one Gram-negative (*Escherichia coli* ATCC 25922) and one Gram-positive (*Staphylococcus aureus* ATCC 25923) bacteria, while the antifungal activity was tested against one yeast (*Candida albicans* ATCC 10231), one filamentous fungus (*Aspergillus fumigatus* ATCC 46645) and three dermatophytes (*Trichophyton rubrum* FF5, *Epidermophyton floccosum* FF9, and *Microsporum canis* FF1). *Candida krusei* ATCC 6258 was used as quality control. The bacteria strains were stored in MHB medium with 15% glycerol at −80 °C and sub-cultured in MHA before each experiment, while the strains of yeast and filamentous fungi were saved in SDB with 20% glycerol at −80 °C and sub-cultured in SDA or PDA before each experiment. All the microorganisms employed in the present study were obtained from the Laboratory of Microbiology, Faculty of Pharmacy, University of Porto (Portugal).

#### 2.8.2. Determination of MIC and MBC/MFC Values

The determination of MIC values of the medicinal mushroom *G. lucidum* was carried out using broth microdilution, described in our previously published research work [33]. In brief, a stock solution of methanolic extract was prepared in dimethyl sulfoxide (DMSO not more than 2% in the final solution prepared), followed by serial dilutions in RPMI (RPMI-1640 with MOPS at pH 7.0) medium for fungi and MHB medium for bacteria to achieve the desired concentration range. Then, 100 µL of each concentration was moved to a well in a 96-well microplate. The inoculum was prepared in physiological water and standardized spectrophotometrically for bacteria and yeast strains, and by spore count for the *A. fumigatus* and dermatophyte strains. Afterwards, 100 µL of the final suspension (1–5 × 10^3^ colony-forming units (CFU)/mL for yeast, 0.4–5 × 10^4^ CFU/mL for *Aspergillus*, 1–3 × 10^3^ CFU/mL for dermatophytes and 1–2 × 10^5^ CFU/mL for bacteria), prepared by dilution in RPMI for fungi and MHB for bacteria, were distributed to each well. Thereafter, the microplates were incubated without agitation at 37 °C for 24 h for *E. coli* and *S. aureus*, for 48 h for *Aspergillus* and *Candida*, and for seven days at 25 °C for the dermatophyte isolates (*T. rubrum*, *E. floccosum*, and *M. canis*). The lowest concentration without visible growth was defined as MIC. The MFC/MBC values were determined by inoculation of 10 µL from wells, showing no turbidity in MIC determination, into a Petri dish of MHA medium for bacteria and into an SDA medium for fungi. The MBC/MFC values were the minimum concentrations which completely inhibited the growth of the microorganisms tested in the same conditions of incubation indicated above. The controls were as follows: sterility with RPMI (for fungi) and with MHB (for bacteria); growth with RPMI and MHB medium plus DMSO (1%) for each, and for fungal and bacterial suspensions, respectively; quality, utilizing *C. krusei* and released with reference voriconazole. Gentamicin, terbinafine and voriconazole were used as positive controls.

### 2.9. Statistical Analysis

All tests were accomplished with three independent samples and each one was done in triplicate. All result values were expressed as mean ± standard deviation (SD), except GC–MS ones. The statistical significance of the data was determined using GraphPad Prism 8.0.1 software (San Diego, CA, USA).

## 3. Results and Discussion

### 3.1. Extraction Yield of Ganoderma lucidum

The extraction of biomolecules from *G. lucidum* was carried out by the maceration method using a shaking water bath, which is ideal for thawing, mixing and shaking samples. This technique obtained an extraction yield of 13.19% of the dry fruiting body used (Table 1). This was similar to that obtained for mushrooms from Turkey mushroom using ethanol (11.7%), while being higher when using methanol as a solvent for extraction in the same study (3.2%) [34], and in previous works from Nigeria (5.30%) [35] and from China using samples from the market (5.61–6.15%) [36]. Additionally, Ćilerdžić et al. reported extraction yields, from three cultivated fruiting bodies of *G. lucidum* from Serbia, China and Montenegro and a commercial strain, using ethanol as solvent, of 6.38–8.85% [37].

### 3.2. Bioactive Compounds Contents of Ganoderma lucidum

The determination of the contents of bioactive compounds in *G. lucidum* fruiting body growing in Morocco was carried out spectrophotometrically, and the results are summarized in Table 1. The results demonstrated that Moroccan *G. lucidum* is an interesting source of bioactive compounds, mainly total phenols and total flavonoids, which correlated well with antimicrobial and antioxidant activities [38]. In addition, the consumption of foods containing high polyphenol content can reduce the risk of heart disease by slowing atherosclerosis progression [39].

The total phenolic content of the methanolic extract of the *G. lucidum* basidiocarps was found to be 154.60 mg of GAE/g of dme. This high value confirmed that phenolics are one of the most interesting bioactive compounds present in mushrooms. Numerous studies from different countries have determined the total phenolic contents in wild, cultivated and commercialized *G. lucidum* basidiocarps, using various solvents (butane, chloroform, ethanol, ethyl acetate, methanol, n-hexane, petroleum ether, and water). Most of these studies recorded lower amounts than those of our samples, with a value ranging from 9.09 to 139 mg GAE/g of the dry extract [34,36,37,40,41,42,43,44,45,46,47,48]. On the contrary, the ethyl acetate extract from Algerian wild mushroom dry extracts (171 mg/g) [46] and methanolic (281.27 mg/g), ethanol (301 mg/g) and water (360.62 mg/g of dry extract) and extracts from Pakistani samples [47], using gallic acid as standard, contained higher contents than those obtained from Moroccan *G. lucidum* methanolic extract.

The result of total flavonoid content determination indicated that *G. lucidum* growing in Morocco is a rich source of this important antioxidant compound with a value of 60.55 mg of CEs/g of dme, which was quite a lot higher than that found in the Algerian study [46]. Two studies from Pakistan and Turkey reported high amounts of total flavonoids in wild *G. lucidum*, with values of 73.02–217 mg CEs/g in the dry extract [42,47]. These results were unlike several previous investigations, which recorded low contents (less than 18 mg/g of dry extract) of total flavonoids in different solvent extracts, from different sample origins, and with the different standards used for the calibration curves [34,37,41,45].

The content of ascorbic acid was estimated and expressed in terms of a milligram of l-ascorbic acid per gram of dry weight (AAE/g dw). The ascorbic acid content in *G. lucidum* was observed to be moderate, with a value of 4.69 mg AAE/g of dw, which was similar to the study from Turkey (4.33 mg/g) [42]. Mau et al. did not detect ascorbic acid in two types of *G. lucidum* from Taiwan [35], while the samples from Turkey and Pakistan showed higher amounts than our sample in different solvent extracts [44,47]. This result confirmed ascorbic acid as being the simplest vitamin found in mushrooms, thought to exert a protective role against several oxidative stress-related diseases, including cancer, stroke, heart disease, and several neurodegenerative diseases [49].

Concerning total tannin and carotenoid contents, *G. lucidum* contained less than for the bioactive compounds above. The fruiting bodies had 2.42 mg CE/g of dw of total tannin content. In regard to carotenoids, β-carotene content was observed to be double that of lycopene in methanolic extract, with values of 0.24 and 0.11 mg/g of dme, respectively. In comparing our carotenoid contents with previous values reported by Sharif et al. [47] and Celik et al. [34], our β-carotene content was quite similar to those found in several extracts. Our lycopene content was higher than those of the previous studies mentioned. β-carotene was not detected in methanolic extract in studies by Tel et al. [42] and Mau et al. [36] while, in a study by Tel et al., higher lycopene (0.67 mg/g) was reported [42]. On the other hand, Sharif et al. detected tannin in methanolic and water extracts, which was absent in ethanol, ethyl acetate and *n*-hexane extracts [47]. Tannin acts as an antioxidant like other polyphenol compounds. Carotenoids are considered to be key antioxidants found in mushrooms after polyphenols, tocopherols and ascorbic acids [49].

Overall, the results demonstrated that *G. lucidum* growing in Moroccan forests is an important and rich source of bioactive compounds, offering many human health benefits. Finally, we conclude that the contents of the bioactive compounds can be affected by many factors, such as time, temperature, solvent, method of extraction, and basidiocarp source.

### 3.3. Biomolecules of Ganoderma lucidum by GC–MS Analysis

**Figure 1:**
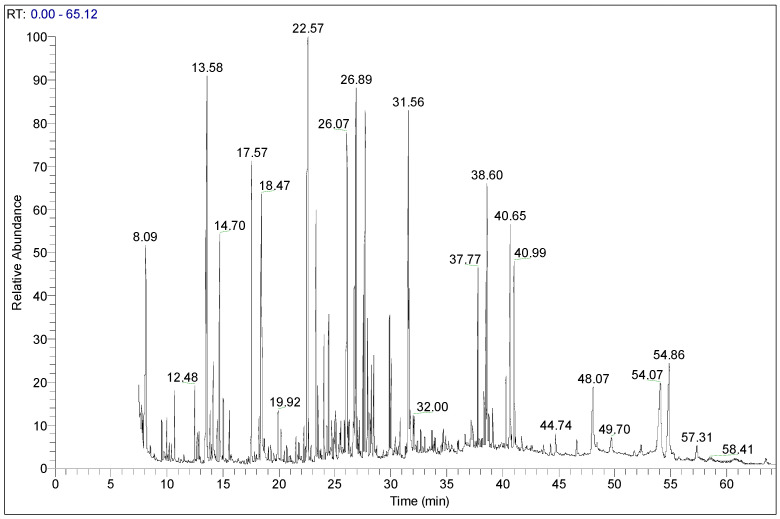
GC–MS chromatogram of *G. lucidum* derivatized methanolic extract.

The identification of biomolecules existing in *G. lucidum* was carried out via GC–MS analysis, a powerful technique for the detection and identification of many substances [50]. In Figure 1, the GC–MS chromatogram of the analyzed extract illustrates numerous peaks, indicating the presence of eighty biologically active compounds, mainly divided into the following major groups: sugars (49.49%), organic acids (8.89%), fatty acids (7.75%), amino acids (7.44%), steroids (7.32%), polyphenols (5.92%) and the rest of the constituents representing 13.16% of the total (99.97%) (Table 2 and Tables S1–S7). The main components obtained were xylitol (7.69%), phosphoric acid (5.70%), galactitol (5.56%), α-D-allopyranose (5.28%), turanose (5.25%) and linoleic acid (5.13%).

The *G. lucidum* methanolic was composed of thirty sugars, representing half (49.49%) of the constituents identified in the sample. Most sugars detected were classed as monosaccharides (39.06%), while the rest were disaccharides (10.43%). Xylitol (7.69%), galactitol (5.56%), α-D-allopyranose (5.28%), turanose (5.25%) and α-D-Talopyranose (4.59%) were the main sugar compounds determined (Table S1). The main compound, xylitol, which was also identified in the sample from Thailand [51], is a sugar alcohol, well known as an anticaries agent and reported to act as an anti-inflammatory and antimicrobial agent [52]. The sugar compositions of the Moroccan sample were observed to be rich and diverse. However, a few sugar compounds were identified in several previous works, including trehalose, fructose, sucrose, glucose, mannitol [43,48,51,53], xylose, mannose [51,54], rhamnose [48,54], fucose, n-acetylglucosamine [54] and cellobiose [51]. In addition, Taofiq et al. indicated that fructose was the only one identified in the sample from Portugal [54].

Concerning organic acids (Table 2 and Table S2), the extract contained ten molecules representing 8.89%, identifying the second group of biomolecules. The most abundant organic acids identified in the analyzed methanolic extract of *G. lucidum* were malic acid (3.64%), fumaric acid (1.89%) and citric acid (1.31%). These compounds, and other organic acids, are known for their antioxidant activities, which may have a protective role against numerous diseases [55]. There are only two published works on the organic acids of *G. lucidum*, a study by Obodai et al., which quantified oxalic acid, malic acid and fumaric acid in the extract from Ghana [48], and a study by Stojković et al., which revealed five organic acids, three of which (oxalic, quinic, and malic acids) were detected in both samples from Serbia and China, unlike, citric and fumaric acids, found only in the Serbian study [53].

Regarding fatty acids (Table 2 and Table S3), the extract was composed of eight compounds (7.75%), predominantly by linoleic acid (5.10%), a polyunsaturated essential fatty acid previously reported to act as an antioxidant [56], and as an inductor of blood pressure in regard to cardiovascular diseases and arthritis, as well as being a minimizer of triglyceride levels [48]. Similar to our results, three previous studies recorded that linoleic acid, palmitic acid, oleic acid, and stearic acid were the main fatty acids identified in *G. lucidum* fruiting bodies [48,53,57].

Similar to fatty acid, the methanolic extract of *G. lucidum* contained eight amino acids (7.44%) (Table 2), wherein pidolic acid (also known as pyroglutamic acid) was the most abundant metabolite detected, with a percentage of 4.72% (Table S4). Recently, Šudomová et al. indicated that pyroglutamic acid revealed anti-urease activities, anti-phosphodiesterase type 5 and anti-angiotensin-converting enzyme [58]. Zhang et al. [59] and Wang et al. [54] identified 16 and 18 amino acids in their samples, leucine and glutamic acid being the most abundant compounds, respectively. In addition, Zhang et al. reported that an amino acid extract showed robust antidiabetic and antioxidant activities [59].

Concerning steroids, over 20 substances have been reportedly identified in *G. lucidum* and their structures listed in cholesterols and ergosterols [2]. However, four compounds were detected in our sample with a percentage of 7.32%, these being ergosterol (3.55%) and ergosta-7,22-dien-3β-ol (3.02%), the major sterol identified (Table S5). Generally, ergosterol and other steroids have been reported to have several biological properties, such as antioxidant, antimicrobial, anti-inflammatory, and anticancer properties, as well as preventing of common diseases [60,61,62].

GC–MS analysis of *G. Lucidum* extract identified six polyphenols and fourteen compounds belonging to different groups (Table 2). The polyphenols were predominantly benzene, (3-chloro-1-propenyl)- (3.79%), gentisic acid (0.65%) and pyrogallol (0.49%) (Table S6), while, phosphoric acid (5.70%), glycerol-3-phosphate (2.63%) and prostaglandin D₂ (2.19%) were the most abundant compounds in the rest of the biomolecule groups (Table S7).

Alongside nutritional and nutraceutical values, the biomolecules identified in wild *G. lucidum* fruiting bodies from Morocco have many other health benefits.

### 3.4. Individual Phenolic Compounds of Ganoderma lucidum by HPLC–MS Analysis

**Figure 2:**
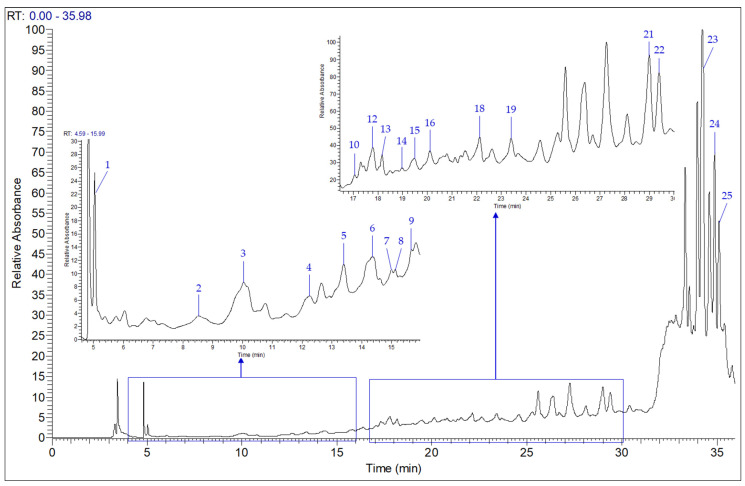
HPLC–MS chromatogram of individual polyphenols in *G. lucidum* extract detected at 280 nm.

Characterization of individual polyphenols in the *G. lucidum* basidiocarps was carried out using the HPLC–MS technique. Figure 2 shows the HPLC–MS chromatogram at 280 nm, illustrating phenolic compound peaks, while the contents of quantified compounds are summarized in Table 3. The HPLC–MS analysis detected many polyphenols in Moroccan *G. lucidum*. Among them, twenty-two phenolic acids, flavonoids and related compounds were identified and quantified, based on commercial standards and their UV, mass spectra and retention times. Isorhamnetin (3561 µg/g of dw), apigenin (1955 µg/g), kaempferol (1714 µg/g) and quercetin (947.2 µg/g) were the major flavonoids quantified in the phenolic extract, followed by chlorogenic acid (442.90 µg/g), which classified as the main phenolic acid determined (Figure S1). The least identified polyphenols were syringic and vanillic acids, with quantities of 3.79 and 14.05 µg/g of dw, respectively. Luteolin, luteolin 7-glucoside and rosmarinic acid were not detected. The results showed that the contents obtained for the flavonoid class were higher than those for phenolic acids and related molecules, which was in agreement with a recent study on Polish Reishi by Kolniak-Ostek et al. [63]. In addition, only apigenin and quercetin phenolic compounds were found in the two studies, with a higher amount in our sample than in the Polish one.

Concerning other published works, three studies analyzed the phenolic compounds of *G. lucidum* growing wild in Portugal. Heleno et al. [43] and Reis et al. [64] reported methanolic extracts containing *p*-hydroxybenzoic, *p*-coumaric and cinnamic acids in low contents, while, in the third study, *p*-hydroxybenzoic, protocatechuic and syringic acids were quantified in the ethanolic extract with concentrations of 2980, 1807 and 1510 µg/g, respectively. Stojković et al. found protocatechuic and cinnamic acids in samples from Serbia and China, while *p*-coumaric and *p*-hydroxybenzoic acids were only detected in China and Serbia, respectively [53]. In addition, these previous four compounds were also quantified in *G. lucidum* extracts from Ghana [48]. On the other hand, Veljović et al. [40] detected gallic acid and *trans*-cinnamic acid (phenolic acids), and quercetin, kaempferol, hesperetin and naringenin (flavonoids) in their ethanolic extracts in lower amounts than in our sample. However, the presence of hesperetin and naringenin were not analyzed in the current study. Finally, *G. lucidum* from Korea was analyzed for 30 phenolic compounds, and among them, only twelve were detected and quantified, of which quercetin, kaempferol and myricetin were the predominant compounds [65]. Based on the literature, chlorogenic acid, catechin, caffeic acid, vanillic acid, rutin, ellagic acid, vanillin, ferulic acid, apigenin 7-glucoside, salicylic acid, methylparaben, kaempferol and isorhamnetin were detected for the first time in *G. lucidum* fruiting body (Figure S1).

Generally, besides the phenolic acids, the present results confirmed that mushrooms are also a rich flavonoid source. Numerous studies on mushrooms have proven that phenolic compounds possess strong biological activities, including antioxidant, antimicrobial, anti-inflammatory and anti-tumor properties [49,66,67,68].

### 3.5. Antioxidant Properties of Ganoderma lucidum Methanolic Extracts

Antioxidants are necessary substances in the human body to balance out free radicals related to numerous chronic health problems [69]. Antioxidants from natural sources remain the best choice due to their numerous human benefits [70]. Herein, three important spectrophotometric methods were chosen to evaluate the antioxidant capacities of the methanolic extract of *G. lucidum* fruiting body: DPPH, β-carotene/linoleic acid, and ferric ion reducing power assays. The results of antioxidant activity are expressed in EC_50_ values, as presented in Table 4. These values were calculated using graphs illustrated in Figures S2–S4. *G. lucidum* methanolic extract showed strong antioxidant capacities using the three methods, with EC_50_ values ranging between 43.75 and 76.62 µg/mL. The best antioxidant capacity of the *G. lucidum* extract was provided by the β-carotene/linoleic acid assay, while the lowest one was by the ferric ion-reducing power method, with an EC_50_ value of 76.62 µg/mL, which was better than the Trolox (80.11 µg/mL), the standard used as a control. These powerful antioxidant properties of the Moroccan mushroom can be explained by the presence of large amounts of antioxidants, namely, phenolics, flavonoids, carotenoids, organic acids, ascorbic acid, fatty acids and other natural molecules, which are useful against many diseases relating to oxidative stress [28].

Regarding DPPH radical-scavenging activity, the antioxidant activity of *G. lucidum* increased to a concentration of 0.4 mg/mL and reached a plateau of 94.56–96.64% at 0.4–0.8 mg/mL (Figure S2). The EC_50_ value of the extract was 53.70 µg/m, which was higher by about two and a half times that of the reference (19.17 µg/mL). In addition, the values were nearly similar to the values (45.16–55 µg/mL) of *G. lucidum* extract from Pakistan and Turkey, using ethanol, methanol and water solvents for extraction [42,44,47]. On the other hand, the phenolic extract (EC_50_ = 140 µg/mL), in a study by Heleno et al., was less effective at radical-scavenging activity than the current extract [43]. Likewise, our methanolic extract exhibited higher antiradical capacity than those of several studies from different countries, including Portugal, Ghana, Serbia China, Montenegro and Turkey, with EC_50_ values ranging between 0.73 and 7.49 mg/mL [34,37,48,53,57]. Kebaili et al. investigated Algerian *G. lucidum* and found that ethyl acetate fraction (IC_50_ = 28 µg/mL) had better antioxidant properties than butane (61 µg/mL) and chloroform fractions (129 µg/mL) [46]. The ethanolic extract from Philippines fungi gave the best antioxidant activity, with EC_50_ equal to 10.69 µg/mL [71].

The antioxidant capacity of the studied mushroom by means of β-carotene-linoleic acid bleaching activity increased in concentration from 0.025 to 0.4 mg/mL and reached a plateau of 89.97–92.41 % at 0.4–0.8 mg/mL (Figure S3). The EC_50_ value of the extract was 43.75 µg/mL, which was higher than that of synthetic antioxidants (3.84 µg/mL). The methanolic extract of Moroccan mushroom possessed much stronger antioxidant capacity than those from the extracts from Ghana, Turkey, Serbia and China, with EC_50_ values of 900–2200, 123.70, 310 and 220 µg/mL, respectively [42,48,53]. Additionally, Heleno et al. reported lower β-carotene-linoleate bleaching activity (EC_50_ = 260 µg/mL) than our extract using a phenolic extract of Portuguese *G. lucidum* [43].

Concerning ferric ion-reducing power activity, the antioxidants present in the methanolic extract of *G. lucidum* reduced the Fe^3+^/ferricyanide complex to the ferrous form. The extract showed steadily increasing reducing power to 2.37 at 0.4 mg/mL (Figure S4). The methanolic fraction (EC_50_ = 76.62 µg/mL) of the current study showed stronger iron-reducing power than the fractions from Ghana, Serbia and China (EC_50_ values of 240–1070 µg/mL) [48,53], and even higher than the reference standard (EC_50_ = 80.11 µg/mL). Previously, two studies tested the reducing power of *G. lucidum* from Portugal, resulting in EC_50_ values of 150 and 620 µg/mL for ethanolic [57] and phenolic [43] extracts, respectively. Furthermore, Kebaili et al. recorded that ethyl acetate extract was more effective in reducing power than chloroform and butanoic extract of Algerian fungi, with IC_50_ = 22, 85 and 108 µg/mL, respectively [46].

### 3.6. Antimicrobial Properties of Ganoderma lucidum Methanolic Extracts

*G. lucidum* is one of the most extensively studied mushrooms as a functional food and for its medicinal properties, specifically in terms of its polysaccharide and triterpenoid compounds and their antitumor activities [15]. Several studies evaluated *G. lucidum* extracts of different solvents and their bioactive compounds against many fungal strains and showed important antimicrobial properties [72,73]. However, there are only a few works that have been published on the antimicrobial properties against dermatophytes. Herein, one of the main objectives of this study was to investigate the antimicrobial properties of the Moroccan *G. lucidum* methanolic extract against seven human pathogenic microorganisms, including bacteria, yeasts and filamentous fungi, using broth microdilution methods to determine the MIC and MBC/MFC. The results demonstrated that methanolic extracts of the samples inhibited the growth of all microorganisms tested at concentrations varying from 1 to 16 mg/mL (Table 5 and Table 6). Among these significant results, the most sensitive pathogen was *E. floccosum* with MIC and MFC of 1 mg/mL, while *A. fumigatus* was the most resistant microorganism with a MIC equal to 16 mg/mL and MFC ≥ 16 mg/mL. The reference antimicrobial agents used were statistically more effective than the mushroom extract.

The antibacterial property of the *G. lucidum* methanolic extract against the two bacterial strains, *E. coli* and *S. aureus*, was observed to be moderate. *S. aureus* was more sensitive than *E. coli* with MIC values of 4 and 8 mg/mL, respectively, while the MBC values of both strains were equal to 8 mg/mL (Table 5). The antibacterial activity of *G. lucidum* extract has been widely evaluated against *E. coli* and *S. aureus* using different solvents, extraction methods, techniques of evaluation, and either wild or cultivated forms, and the results differ from one study to another, which makes it difficult to compare our results and previously published ones. The Moroccan mushroom extract demonstrated better antibacterial results than several studies, and less important results than others. Hence, concerning the *E. coli* strain, the solvent extracts (chloroform, hexane, methanol and ethyl acetate) from Iran, Nigeria and India, were reported to have no activity [35,74,75,76], while possessing activity in other studies from Nigeria, India, Nepal and Pakistan at a concentration of 20–200 mg/mL, without arriving at MBC values [47,57,77,78,79,80]. Regarding *S. aureus*, the methanolic extracts of *G. lucidum* did not inhibit the microorganisms in studies from India, conducted by Sheena et al. [80], and from Nigeria, conducted by Shamaki and Geidam [35]. However, several *G. lucidum* extracts (chloroform, ethanol, ethyl acetate, methanol, n-hexane and water) showed activity with MIC values of 6.25–100 mg/mL in other previous studies [47,71,74,75,79].

Taofiq et al. found that *G. lucidum* ethanolic extract from the Portuguese market could inhibit the growth of both bacteria *S. aureus* and *E. coli* with MIC of 5 and 10 mg/mL, respectively [57], similar to our results. Likewise, Keypour et al., working on chloroform extract against *S. aureus* using the disk diffusion method, reported an MBC equal to our one (8 mg/mL) [74].

However, Heleno et al. observed that the Portuguese *G. lucidum* phenolic extract presented high potential activity against *S. aureus* and *E. coli* with MICs of 0.025 and 0.35 mg/mL and MBCs of 0.035 and 0.75 mg/mL, respectively, revealing stronger activities than the standards used (ampicillin and streptomycin) [81]. Likewise, other studies on wild and cultivated basidiocarps from countries like Serbia, China, Montenegro, Congo and India, used different solvents (methanol, ethanol, ethyl acetate and chloroform) and techniques to determine MIC and MBC, and also showed important antibacterial activities against *S. aureus* and *E. coli* with MICs of 0.07–2.77 and 0.15–4.07 mg/mL, and MBCs of 0.15–4.07 and 0.30–4.07, respectively [37,53,76,82].

The antifungal properties of *G. lucidum* growing in Morocco were evaluated and the results demonstrated that methanolic extract was active against the five human pathogenic fungi tested, with MIC and MFC values ranging between 1 and 16 mg/mL (Table 6). The yeast *C. albicans* and the filamentous fungus *A. fumigatus* were the most resistant, with an MIC of 16 mg/mL for both, and MFC of 16 and ≥16 mg/mL, respectively. The extract exhibited strong activity against dermatophytes (MICs and MFCs ranging between 1 and 3.33 mg/mL), with *E. floccosum* being the most susceptible fungi, with MIC and MFC equal to 1 mg/mL. When comparing the antifungal results with the published ones, our methanolic extract was better against *C. albicans* than the ethanolic extract of *G. lucidum* from the Portuguese market, with a MIC value superior to 20 mg/mL [57]. Contrarily, the chloroform and hexane extract of the Iranian mushroom inhibited the growth of *C. albicans* at a MIC value of 6.25 mg/mL [75]. For *A. fumigatus*, the methanolic extract for the Indian sample released low activity (50 mg/mL) utilizing the disc diffusion method [83]. The ethanol and methanolic extracts of wild and cultivated basidiocarps from Serbia, China and Montenegro demonstrated an important antifungal activity with MICs of 0.07–1.5 mg/mL and MFCs of 1.25–3.37 mg/mL [37,53]. *A. fumigatus* was one of the three most resistant fungi to *G. lucidum* phenolic extract in a previous work by Helene et al., which presented values of 1.5 and 3 mg/mL for MIC and MFC, respectively, which were better than our extract values [81].

As summarized in Table 6, the methanolic extract of *G. lucidum* growing in the Moroccan forest demonstrated significant antifungal activity against the three tested dermatophytes. Our *G. lucidum* methanolic extract was more effective than the extracts of the Nigerian sample against *E. floccosum* and *T. rubrum*. The ethanolic extract showed activity at 10 and 50 mg/mL presenting 10.60% and 28.80% inhibition, respectively, while petroleum ether extract inhibited the fungi at 20 mg/mL, inhibiting 11.30% of *E. floccosum* and 29% of *T. rubrum* [41]. In addition, our previous published work reported that *Lactarius sanguifluus* methanolic extract was generally less potent against the three dermatophytes than the present extract [33].

Overall, the potent antimicrobial properties found in the current investigation could be related to the many bioactive compounds identified and determined in *G. lucidum* methanolic extract, including polyphenols (total and individual), organic and fatty acids, steroids and many other biomolecules. Finally, these important antimicrobial properties suggest that the extract of *G. lucidum* growing in Morocco could be considered potentially useful for the treatment of many health conditions.

## 4. Conclusions

In this research work, the medicinal mushroom *G. lucidum* growing wild in Moroccan forests was investigated for its biochemical composition and biological activities for the first time, and the results proved that the Moroccan mushroom is a very interesting source of bioactive compounds. In particular, it contained a high quantity of phenolic and flavonoid (total and individual) compounds, as well as being composed of several biologically active molecules grouped mainly in the classes of sugars, organic acids, fatty acids, amino acids and steroids. *G. lucidum* methanolic extract demonstrated strong antioxidant activity and the extract demonstrated potent antimicrobial properties in inhibiting all tested human pathogenic microorganisms, including two bacteria, and five fungi strains. Overall, it can be considered that *G. lucidum* growing in Moroccan forests is a rich source of nutritional, nutraceutical and pharmaceutical compounds, which are responsible for many human health benefits. Moreover, these findings suggest that the Moroccan mushroom could be extremely useful in the food and medicinal industries and could positively affect socioeconomic status.

## Figures and Tables

**Table 1:** Extraction yields and bioactive compound contents in *G. lucidum* basidiocarps ^1^.

Bioactive Compounds	Content
Extraction yield (%)	13.19 ± 0.52
Total phenolic (mg GAE/g dme)	154.60 ± 2.38
Total flavonoid (mg CE/g dme)	60.55 ± 1.74
Total ascorbic acid (mg AAE/g dw)	4.69 ± 0.22
Total tannin (mg CE/g dw)	2.42 ± 0.42
Total β-carotene (mg/g dme)	0.24 ± 0.01
Total lycopene (mg/g dme)	0.11 ± 0.00

^1^ Values are expressed as means ± SD of three independent measurements.

**Table 2:** Groups of biomolecules identified in derivatized extracts of *G. lucidum* by GC–MS analysis.

Biomolecule Groups	Area (%)	Number of Compounds
Sugar compositions	49.49	30
Organic acids	8.89	10
Fatty acids	7.75	8
Amino acids	7.44	8
Steroids	7.32	4
Polyphenols	5.92	6
Others	13.16	14
**Total**	**99.97**	**80**

**Table 3:** Individual polyphenols in the *G. lucidum* basidiocarps characterized by HPLC–MS in negative mode ¹.

Peak N°	Rt (min)	MW	Recorded *m*/*z*	Individual Polyphenols	Content (µg/g dw)
1	5.02	170.02	169.67	Gallic acid	69.11 ± 0.22
2	8.52	154.12	153.40	Protocatechuic acid	36.72 ± 0.28
3	10.06	354.31	353.14	Chlorogenic acid	442.9 ± 1.47
4	12.32	290.08	289.11	Catechin	249.6 ± 1.20
5	13.40	138.03	137.16	*p*-Hydroxybenzoic acid	81.84 ± 0.23
6	14.37	180.04	178.88	Caffeic acid	21.01 ± 0.09
7	15.00	168.04	167.90	Vanillic acid	14.05 ± 0.51
8	15.12	198.05	197.04	Syringic acid	3.79 ± 0.08
9	16.60	610.15	609.98	Rutin	135.6 ± 0.42
10	17.18	302.20	301.22	Ellagic acid	178.3 ± 0.52
11	-	-	-	Luteolin 7-glucoside	ND
12	17.80	164.05	164.97	*p*-Coumaric acid	158.4 ± 0.76
13	18.21	152.05	151.27	Vanillin	75.8 ± 0.36
14	18.96	194.19	193.18	Ferulic acid	40.29 ± 0.42
15	19.44	580.18	178.88	Naringin	140 ± 0.21
16	20.12	432.11	432.02	Apigenin 7-glucoside	427.5 ± 1.18
17	-	-	-	Rosmarinic acid	ND
18	22.15	138.03	137.36	Salicylic acid	376.4 ± 0.56
19	23.31	152.05	151.20	Methyl paraben	301.4 ± 1.36
20	-	-	-	Luteolin	ND
21	29.01	302.04	301.49	Quercetin	947.2 ± 0.56
22	29.35	148.05	148.14	Cinnamic acid	105.5 ± 0.29
23	34.19	270.05	269.22	Apigenin	1955 ± 4.14
24	34.94	286.05	285.79	Kaempferol	1714 ± 6.01
25	35.19	316.27	316.14	Isorhamnetin	3561 ± 6.85

¹ Each value is expressed as means ± SD of three independent measurements. Rt—retention time; ND—not detected; dw—dry weight.

**Table 4:** EC_50_ values (µg/mL) of antioxidant activity of the *G. lucidum* methanolic extracts and Trolox^® 1^.

Assays	*G. lucidum* Extract	Trolox
DPPH radical-scavenging activity	53.7 ± 1.65	19.17 ± 0.99
β-carotene/linoleic acid assay	43.75 ± 1.29	3.84 ± 0.70
Ferric ion-reducing power assay	76.62 ± 0.45	80.11 ± 2.37

^1^ Values are expressed as means ± SD of three independent measurements.

**Table 5:** MIC and MBC values of *G. lucidum* methanolic extract and reference antibacterial agents ^1^.

Microorganisms	*G. lucidum* Extract (mg/mL)		Gentamicin (µg/mL)	
	MIC	MBC	MIC	MBC
*Escherichia coli*	8.00 ± 0.00	8.00 ± 0.00	2.00 ± 0.00	>32
*Staphylococcus aureus*	4.00 ± 0.00	8.00 ± 0.00	0.38 ± 0.14	16.00 ± 0.00

^1^ Each value is expressed as means ± SD (n = 4–6). MIC—minimum inhibitory concentration; MBC—minimum bactericidal concentration.

**Table 6:** MIC and MFC values of *G. lucidum* methanolic extract and reference antifungal agents ^1^.

Microorganisms	*G. lucidum* Extract (mg/mL)		Voriconazole (µg/mL)		Terbinafine (µg/mL)	
	MIC	MFC	MIC	MFC	MIC	MFC
*Candida albicans*	16.00 ± 0.00	16.00 ± 0.00	0.38 ± 0.14	>4	-	-
*Aspergillus fumigatus*	16.00 ± 0.00	≥16	0.25 ± 0.00	0.75 ± 0.29	-	-
*Trichophyton rubrum*	1.50 ± 0.58	2.50 ± 1.73	0.13 ± 0.00	1.50 ± 0.58	0.011 ± 0.005	0.129 ± 0.14
*Epidermophyton floccosum*	1.00 ± 0.00	1.00 ± 0.00	0.03 ± 0.00	0.09 ± 0.04	0.023 ± 0.009	0.038 ± 0.005
*Microsporum canis*	2.00 ± 0.00	3.33 ± 1.03	0.13 ± 0.00	0.38 ± 0.14	0.120 ± 0.100	0.750 ± 0.433

^1^ Each value is expressed as means ± SD (n = 4–6). MIC—minimum inhibitory concentration; MFC—minimum fungicidal concentration.

## Data Availability

Data is contained within the article or Supplementary Material.

## Supplementary Materials

The following are available online at https://www.mdpi.com/article/10.3390/life13051217/s1, Table S1: Sugar compositions of the derivatized methanolic extract of *Ganoderma lucidum* by GC–MS analysis, Table S2: Organic acids of the derivatized methanolic extract of *G. lucidum* by GC–MS analysis, Table S3: Fatty acids of the derivatized methanolic extract of *G. lucidum* by GC–MS analysis, Table S4: Amino acids of the derivatized methanolic extract of *G. lucidum* by GC–MS analysis, Table S5: Steroids of the derivatized methanolic extract of *G. lucidum* by GC–MS analysis, Table S6: Polyphenols of the derivatized methanolic extract of *G. lucidum* by GC–MS analysis, Polyphenols of the derivatized methanolic extract of *G. lucidum* by GC–MS analysis, Table S7: Other biomolecules group of the derivatized methanolic extract of *G. lucidum* by GC–MS analysis, Figure S1: ESI–MS^n^ spectrum of the most important phenolic compounds identified in G. lucidum, Figure S2: Radical-scavenging activity on DPPH radicals. Each value is expressed as mean ± SD (n = 3), Figure S3: Lipid peroxidation inhibition measured by the β-carotene bleaching inhibition. Each value is expressed as mean ± SD (n = 3), Figure S4: Reducing power. Each value is expressed as mean ± SD (n = 3).
