# Supplementary material for: Chemical Characterization and Evaluation of Antimicrobial Properties of the Wild Medicinal Mushroom Ganoderma lucidum Growing in Northern Moroccan Forests
Source: Life (Basel). 2023 May 19;13(5):1217. doi: 10.3390/life13051217 (PMC10223331; doi:10.3390/life13051217)
Supplement: Supplementary file 1 [file life-13-01217-s001.zip › life-2343933-supplementary.pdf]

life

Supplementary File

Chemical Characterization and Evaluation of Antimicrobial Properties of the Wild Medicinal Mushroom *Ganoderma lucidum* Growing in Northern Moroccan Forests.

El Hadi Erbiai [1,2], Benoutman Amina [1], Abbassi Kaoutar [1], Rabah Saidi [1], Zouhaire Lamrani [1], Eugénia Pinto [3,4], Joaquim C.G. Esteves da Silva [2], Abdelfettah Maouni [1], and Luís Pinto da Silva [2*]

[1] Biology, Environment, and Sustainable Development Laboratory, ENS, Abdelmalek Essaadi University, 93000 Tetouan, Morocco; elhadi.erbiai@etu.uae.ac.ma (E.H.E.); amina.benoutman@etu.uae.ac.ma; kaoutar.abbassi@etu.uae.ac.ma; r.saidi@uae.ac.ma (R.S.); zh.amrani@yahoo.fr (Z.L.); amaouni@uae.ac.ma (A.M.)

[2] Chemistry Research Unit (CIQUP), Institute of Molecular Sciences (IMS), Department of Sciences, Environment and Territorial Planning, Faculty of Sciences, University of Porto, Rua do Campo Alegre s/n, 4169-007 Porto, Portugal; el.erbiai@fc.up.pt (E.H.E); jcsilva@fc.up.pt (J.C.G.E.d.S.)

[3] Laboratory of Microbiology, Biological Sciences Department, Faculty of Pharmacy, University of Porto (FFUP), 4050-313 Porto, Portugal; epinto@ff.up.pt (E.P.)

[4] Interdisciplinary Centre of Marine and Environmental Research (CIIMAR), University of Porto, 4450-208 Matosinhos, Portugal

* Correspondence: luis.silva@fc.up.pt (L.P.d.S.)

Citation: Erbiai, E.H.; Amina, B.; Kaoutar, A.; Saidi, R.; Lamrani, Z.; Pinto, E.; Esteves da Silva, J.C.G.; Maouni, A.; Pinto da Silva, L. Chemical Characterization and Evaluation of Antimicrobial Properties of the Wild Medicinal Mushroom *Ganoderma lucidum* Growing in Northern Moroccan Forests. *Life* **2023**, *13*, 1217. https://doi.org/10.3390/life13051217

Academic Editor: Stefania Lamponi

Received: 29 March 2023
Revised: 15 May 2023
Accepted: 17 May 2023
Published: 19 May 2023

Copyright: © 2023 by the authors. Submitted for possible open access publication under the terms and conditions of the Creative Commons Attribution (CC BY) license (https://creativecommons.org/licenses/by/4.0/).

Table S1: Sugar compositions of the derivatized methanolic extract of *Ganoderma lucidum* by GC–MS analysis.

Compound names	Chemical formula	Molecular weight	Kovats' RI*	Area %
1,5-Anhydrohexitol	$C_6H_{12}O_5$	164.16	1989.85	1.52
1-Deoxy-d-ribitol	$C_5H_{12}O_4$	136.15	1533.83	0.35
D-Allofuranose	$C_6H_{12}O_6$	180.16	1996.64	1.19
D-Arabinitol	$C_5H_{12}O_5$	152.15	1710.45	0.28
D-Erythrofuranose (isomer 2)	$C_4H_8O_4$	120.10	1833.33	0.27
D-Fructofuranose (isomer 1)	$C_6H_{12}O_6$	180.16	2155.17	0.69
D-Gluconic acid	$C_6H_{12}O_7$	196.16	2017.01	0.79
D-Glucopyranosiduronic acid	-	-	3090.24	0.33
DL-Arabinopyranose	$C_5H_{10}O_5$	150.13	1993.62	0.22
D-Ribofuranose (isomer 1)	$C_5H_{10}O_5$	150.13	2665.19	0.52
D-Ribono-1,4-lactone	$C_5H_8O_5$	148.11	1634.80	0.13
D-Talofuranose (isomer 2)	$C_6H_{12}O_6$	180.16	1788.03	0.24
**Galactitol**	$\mathbf{C_6H_{14}O_6}$	**182.17**	**1991.19**	**5.56**
Gluconolactone	$C_6H_{10}O_6$	178.14	1777.31	0.32
Glyceric acid	$C_3H_6O_4$	106.08	1318.28	0.27
Glycerol	$C_3H_8O_3$	92.09	1260.23	2.31
Lactulose (isomer 2)	$C_{12}H_{22}O_{11}$	342.30	2777.84	2.69
L-Arabinose	$C_5H_{10}O_5$	150.13	1781.42	0.34
L-Sorbose	$C_6H_{12}O_6$	180.16	2010.31	0.35
Methyl alpha-D-glucofuranoside	$C_7H_{14}O_6$	194.18	2736.36	0.93
Myo-Inositol	$C_6H_{12}O_6$	180.16	1952.72	2.26
N-Acetyl-D-galactosamine (isomer 2)	$C_8H_{15}NO_6$	221.21	2109.77	0.82
Threonic acid	$C_4H_8O_5$	136.10	1538.81	0.11
Trehalose	$C_{12}H_{22}O_{11}$	342.30	2634.18	2.49
**Turanose**	$\mathbf{C_{12}H_{22}O_{11}}$	**342.30**	**2627.85**	**5.25**
**Xylitol**	$\mathbf{C_5H1_2O_5}$	**152.15**	**1726.87**	**7.69**
**α-D-Allopyranose**	$\mathbf{C_6H_{12}O_6}$	**180.16**	**1998.07**	**5.28**
α-D-Glucopyranose	$C_6H_{12}O_6$	180.16	1986.33	0.76
α-D-Mannopyranose	$C_6H_{12}O_6$	180.16	1650.36	0.94
**α-D-Talopyranose**	$\mathbf{C_6H_{12}O_6}$	**180.16**	**2080.54**	**4.59**

*Kovats' RI: Kovats retention index of compound's derivative form.

Table S2: Organic acids of the derivatized methanolic extract of *G. lucidum* by GC–MS analysis.

Compound names	Chemical formula	Molecular weight	Kovats' RI*	Area %
3-Hydroxyisobutyric acid	$C_4H_8O_3$	104.10	1146.99	0.06
Carbamic acid	CH_3NO_2	61.04	1024.84	0.27
**Citric acid**	$\mathbf{C_6H_8O_7}$	**192.12**	**1820.71**	**1.31**
**Fumaric Acid**	$\mathbf{C_4H_4O_4}$	**116.07**	**1329.57**	**1.89**
Glycolic acid	$C_2H_4O_3$	76.05	1066.46	0.12
Lactic Acid	$C_3H_6O_3$	90.08	1053.42	0.2
**Malic acid**	$\mathbf{C_4H_6O_5}$	**134.09**	**1479.08**	**3.64**
Phthalic acid	$C_8H_6O_4$	166.13	2541.45	0.42
Pyruvic acid	$C_3H_4O_3$	88.06	1079.50	0.11
Succinic acid	$C_4H_6O_4$	118.09	1298.86	0.87

*Kovats' RI: Kovats retention index of compound's derivative form.

Table S3: Fatty acids of the derivatized methanolic extract of *G. lucidum* by GC–MS analysis.

Compound names	Chemical formula	Molecular weight	Kovats' RI*	Area %
10,12-Docosadiynedioic acid	$C_{22}H_{34}O_4$	362.50	2813.30	0.11
2-Bromosebacic acid	$C_{10}H_{17}BrO_4$	281.14	2954.77	0.30
2-Hydroxyglutaric acid	$C_5H_8O_5$	148.11	1563.68	0.12
**Linoleic acid**	$\mathbf{C_{18}H_{32}O_2}$	**280.45**	**2197.13**	**5.13**
Oleic acid	$C_{18}H_{34}O_2$	282.46	2205.92	0.46
Palmitic acid	$C_{16}H_{32}O_2$	256.42	2027.84	0.92
Pentadecanoic acid	$C_{15}H_{30}O_2$	242.40	1988.93	0.36
Stearic acid	$C_{18}H_{36}O_2$	284.48	2223.08	0.35

*Kovats' RI: Kovats retention index of compound's derivative form.

Table S4: Amino acids of the derivatized methanolic extract of *G. lucidum* by GC–MS analysis.

Compound names	Chemical formula	Molecular weight	Kovats' RI*	Area %
Alanine	$C_3H_7NO_2$	89.09	1093.79	0.44
Aspartic acid	$C_4H_7NO_4$	133.10	1512.94	0.50
Glutamic acid	$C_5H_9NO_4$	147.13	1610.29	0.23
Leucine	$C_6H_{13}NO_2$	131.17	1282.95	0.43
**Pidolic acid**	$\mathbf{C_5H_7NO_3}$	**129.11**	**1524.38**	**4.72**
Serine	$C_3H_7NO_3$	105.09	1347.85	0.38
Threonine	$C_4H_9NO_3$	119.12	1376.34	0.32
Valine	$C_5H_{11}NO_2$	117.15	1205.11	0.42

*Kovats' RI: Kovats retention index of compound's derivative form.

Table S5: Steroids of the derivatized methanolic extract of *G. lucidum* by GC–MS analysis.

Compound names	Chemical formula	Molecular weight	Kovats' RI*	Area %
16α-Hydroxypregnenolone	$C_{21}H_{32}O_3$	332.50	3027.53	0.23
**Ergosta-7,22-dien-3β-ol**	$\mathbf{C_{28}H_{46}O}$	**398.70**	**3260.87**	**3.02**
Ergosta-7-en-3beta-ol	$C_{28}H_{48}O$	400.70	3342.53	0.52
**Ergosterol**	$\mathbf{C_{28}H_{44}O}$	**396.65**	**3242.78**	**3.55**

*Kovats' RI: Kovats retention index of compound's derivative form.

Table S6: Polyphenols of the derivatized methanolic extract of *G. lucidum* by GC–MS analysis.

Compound names	Chemical formula	Molecular weight	Kovats' RI*	Area %
1,1-Dichloro-2,2-bis(4-methoxyphenyl)ethane	$C_{16}H_{16}C_{12}O_2$	311.2	1217	0.22
**Benzene, (3-chloro-1-propenyl)-**	$\mathbf{C_9H_9Cl}$	**152.621**	**934.18**	**3.79**
Flavone, 5,7-dihydroxy-6c-glucoside	$C_{27}H_{30}O_{14}$	578.5	3128.57	0.46
Gentisic acid	$C_7H_6O_4$	154.1201	1772.14	0.65
Phenol	C_6H_6O	94.11	919.62	0.31
Pyrogallol	$C_6H_6O_3$	126.11	1597.01	0.49

*Kovats' RI: Kovats retention index of compound's derivative form.

Table S7: Other biomolecules group of the derivatized methanolic extract of *G. lucidum* by GC–MS analysis.

Compound names	Chemical formula	Molecular weight	Kovats' RI*	Area %
1,5-Pentanediol	$C_5H_{12}O_2$	104.15	1225.57	0.21
2,4-Dimethylthiazole	$C_5H_7N_s$	113.18	897.42	0.03
2-Monoolein	$C_{21}H_{40}O_4$	356.50	2934.44	0.09
3-Methoxypentane	$C_6H_{14}O$	102.17	1730.35	0.26
3-Methyl-5-phenyl-1H-pyrazole	$C_{10}H_{10}N_2$	158.20	2210.06	0.22
4-Pyridinol	$C_5H_5N_o$	95.10	1037.27	0.11
6-Undecanol	$C_{11}H_{24}O$	172.31	2616.46	0.44
Adenosine	$C_{10}H_{13}N_5O_4$	267.24	2644.30	0.31
Deanol	$C_4H_{11}NO$	89.14	911.39	0.67
Glycerol monostearate	$C_{21}H_{42}O_4$	358.60	2764.77	0.15
**Glycerol-3-phosphate**	$\mathbf{C_3H_9O_6P}$	**172.07**	**1764.68**	**2.63**
Guanosine	$C_{10}H_{13}N_5O_5$	283.24	2785.80	0.15
**Phosphoric acid**	$\mathbf{H_3PO_4}$	**97.99**	**1267.05**	**5.7**
**Prostaglandin D_2**	$\mathbf{C_{20}H_{32}O_5}$	**352.47**	**3078.05**	**2.19**

*Kovats' RI: Kovats retention index of compound's derivative form.

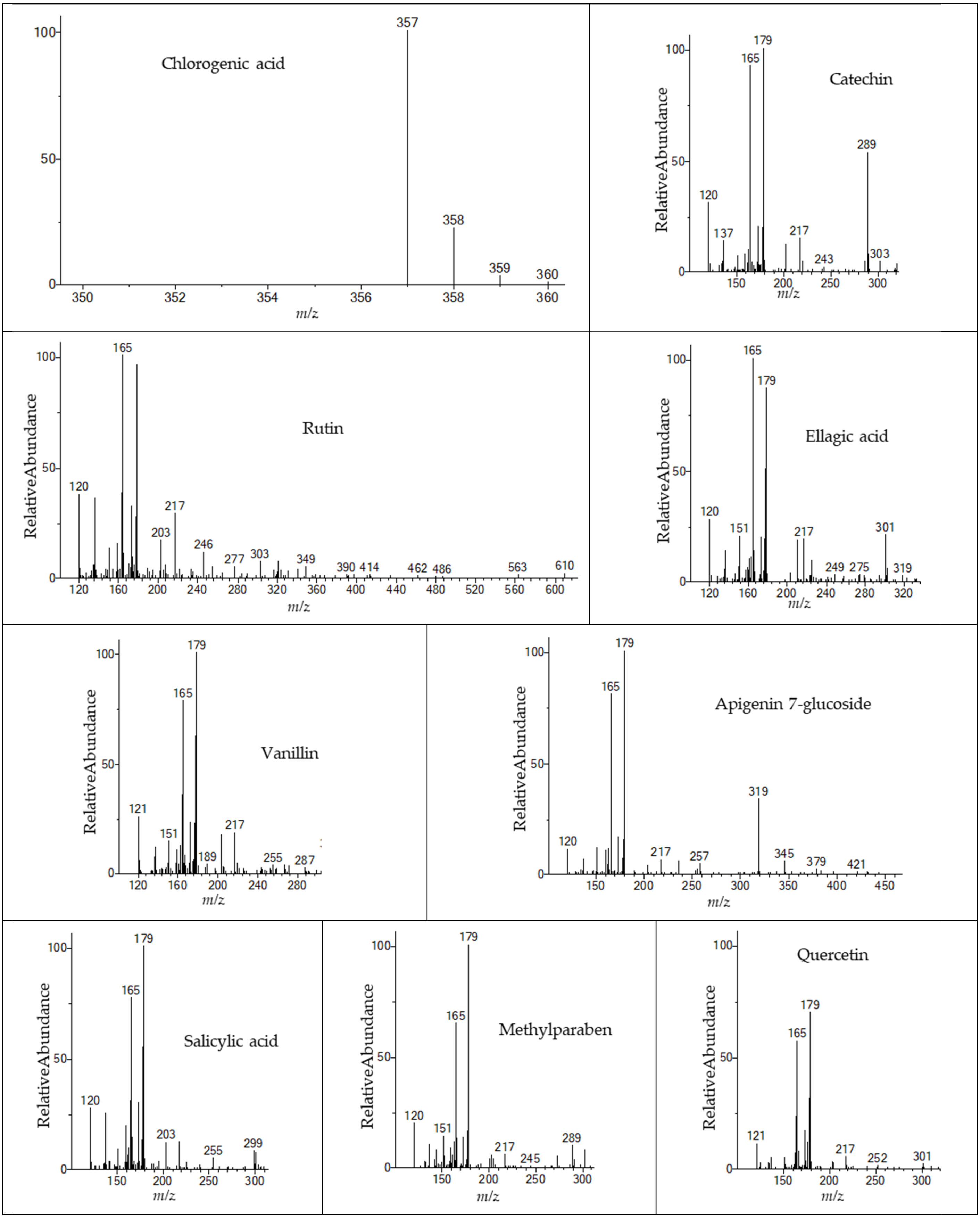

Figure S1: ESI–MS^n spectrum of the most important phenolic compounds identified in *G. lucidum*: Chlorogenic acid at *m/z* 353.14, catechin at *m/z* 289.11, rutin at *m/z* 609.98, ellagic acid at *m/z* 301.22, vanillin at *m/z* 151.27, apigenin 7-glucoside at *m/z* 432.02, salicylic acid at *m/z* 137.36, methylparaben at *m/z* 151.20 and quercetin at *m/z* 301.49.

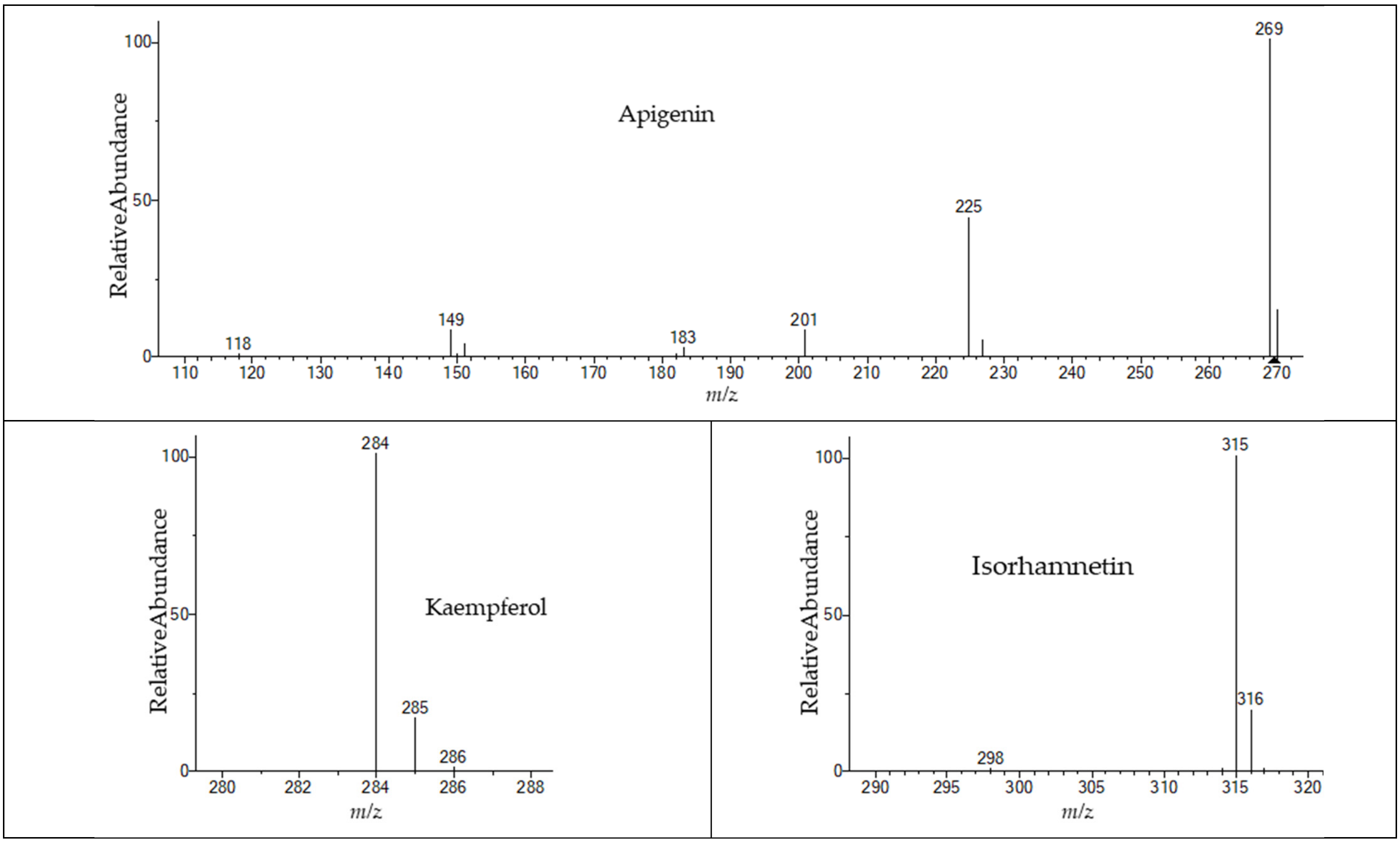

Figure S1 (continued): ESI–MS^n spectrum of the most important phenolic compounds identified in *G. lucidum*: apigenin at *m*/*z* 269.22, kaempferol at *m*/*z* 285.79, and Isorhamnetin at *m*/*z* 316.14.

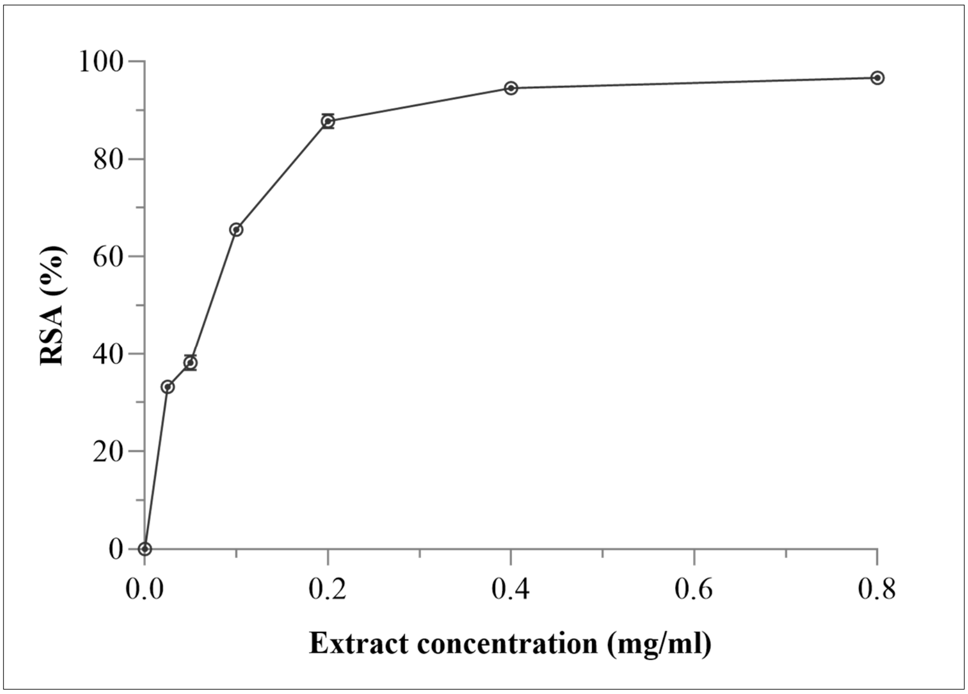

Figure S2: Radical-scavenging activity on DPPH radicals. Each value is expressed as mean ± SD (n = 3).

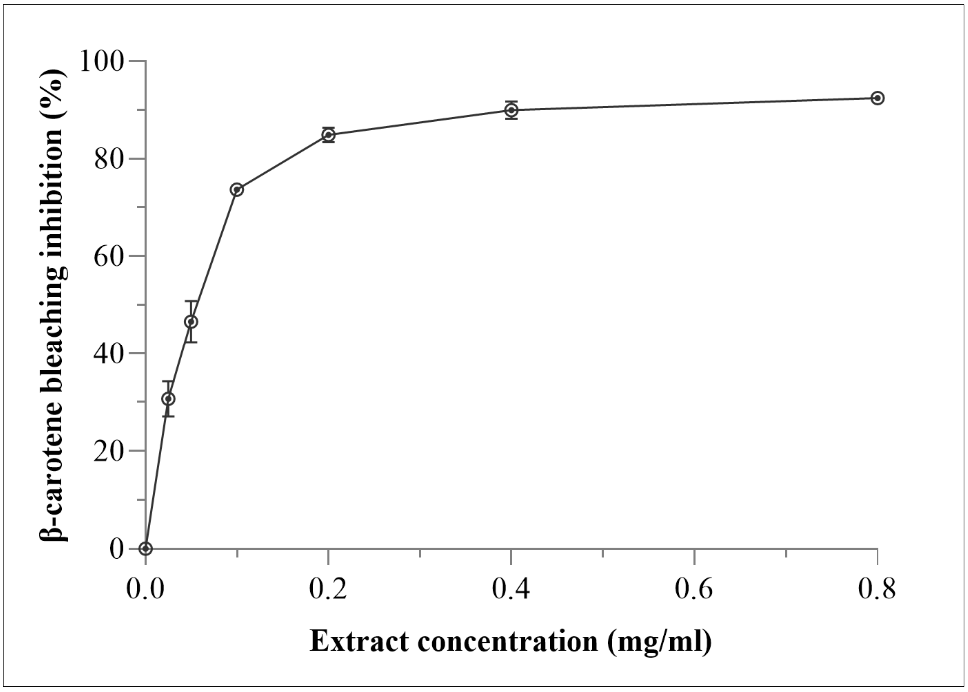

Figure S3: Lipid peroxidation inhibition measured by the β-carotene bleaching inhibition. Each value is expressed as mean ± SD (n = 3).

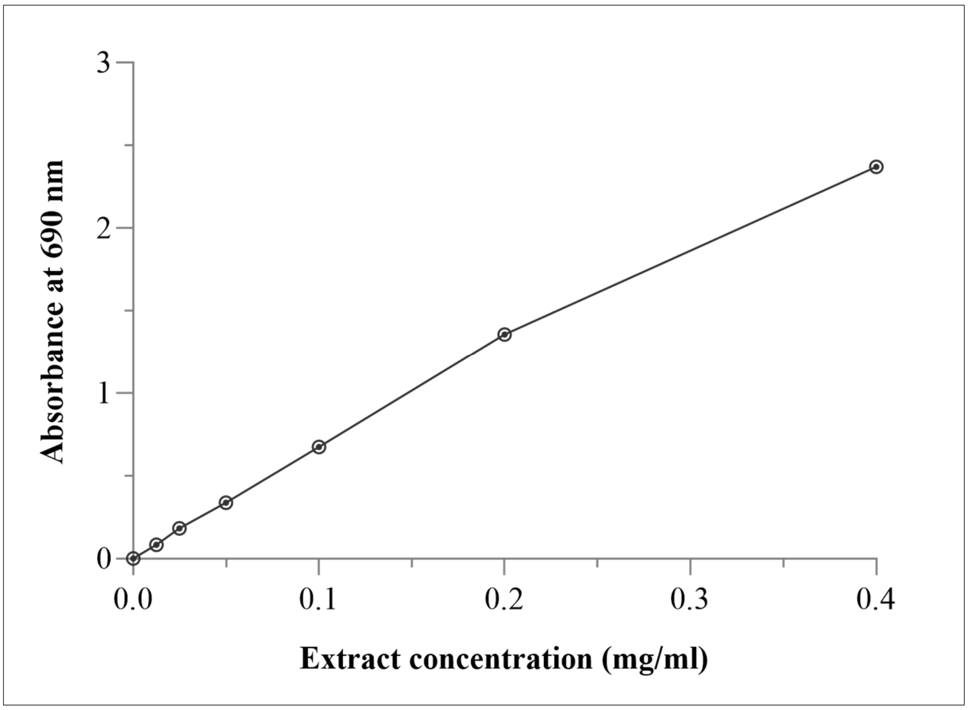

Figure S4: Reducing power. Each value is expressed as mean ± SD (n = 3).
